# Does the surgeon matter in the management of ovarian cancer?

**DOI:** 10.1038/sj.bjc.6690550

**Published:** 1999-07

**Authors:** K K Chan

**Affiliations:** Birmingham Women's Hospital, Metchley Park Road, Edgebaston, B15 2TT, UK


*British Journal of Cancer* (1999) **80**(10), 1492–1493
© 1999 Cancer Research Campaign
Article no. bjoc.1999.0550

Editorial

### Does the surgeon matter in the management of ovarian cancer?

**KK Chan**

Birmingham Women's Hospital, Metchley Park Road, Edgebaston B15 2TT, UK

The survival of patients with ovarian cancer in the UK compares poorly with other Western countries. A large part of this difference must be due to the organization of treatment as well as the treatment given to these patients. For example, Paclitaxel is still not available to all patients with ovarian cancer in the UK, despite the fact that it has been clearly demonstrated that the combination of this drug with Cis-platinum is clearly superior to the combination of platinum analogues with alkylating agents which was the previous gold standard (Hawkins, 1998; Gore, 1998; Sandercock, 1998). The formal comparison of taxol-carboplatin with carboplatin (ICON3) is eagerly awaited (Hawkins, 1998; Sandercock, 1998; Gore, 1998). Although over 80% of patients with ovarian cancer present with advanced disease, the quality of chemotherapy treatment is not the sole factor in determining the success of treatment. There is evidence to support the idea that the surgeon may have an important role to play.

Junor et al (1994) showed an improved survival for patients with ovarian cancer treated by a multidisciplinary team in Scotland. In a national survey of ovarian cancer treated in the USA, Nguyen et al (1993) showed a significantly better survival in those patients operated on by gynaecologists compared with those by general surgeons. Mayer et al (1992) showed a significant improvement in survival for those patients with stage I and II disease staged by a gynaecological oncologist when compared with those patients staged by a non-oncologist. Five-year actuarial and disease-free survival were 83 ± 7% and 76 ± 8% for those staged by a gynaecological oncologist compared to 59 ± 11% ($P < 0.05$) and 39 ± 11% ($P < 0.03$) for the group staged by a non-oncologist.

The better results obtained by gynaecological oncologists and those working in multidisciplinary teams are likely to be the result of more careful staging and a stricter adherence to surgical guidelines. Sengupta's audit of the surgical management of patients referred to a regional oncology centre demonstrates the poor compliance with management guidelines published by the Standing Subcommittee on Cancer of the Standing Medical Advisory Committee in July 1991 (Scott, 1991). These guidelines have become known as the Scott Report[4] and represent a balanced view of the management of ovarian cancer at that time. The majority of the patients (92%) were seen in the out-patient clinic so the operations were elective, yet fewer than half the patients underwent the recommended surgical procedures. Even the recording of surgical details was poor; only in 75 out of 85 case notes inspected was the initial incision recorded. The residual disease status was only recorded in 45 cases despite the well-known prognostic significance of this factor.

Staging is most important in those patients suspected to have stage I disease. Faught et al[5] showed the value of identifying true stage IA or IB patients since these women require surgery only. Schueler et al (1998) performed a comprehensive staging procedure on all their patients and found that 13 out of 45 patients (29%) were upstaged due to the presence of microscopic disease. The complication rate in secondary staging procedures was significantly higher than in primary procedures (77% vs 23%) ($P < 0.05$). In Sengupta's series none of the 21 patients with suspected stage I disease had a staging procedure that followed the Scott Report guidelines fully. None of the women had any lymph node biopsy. Petru et al (1994) found lymph node metastases in nine out of 40 (23%) apparent stage I patients.

Sengupta's audit revealed the varying standards of compliance with the Calman–Hine Report's (Department of Health, 1995) recommendations. In one hospital a single consultant operated on 11 patients, while in another seven consultants operated on 17 patients. The evidence shows that patients with ovarian cancer benefit from undergoing their treatment in multidisciplinary teams and specialized units. It also shows that the specialty of the surgeon performing the initial laparotomy has a direct bearing on the prognosis of the patient. The Calman–Hine Report was published in 1995. It is high time that Health Authorities and Health Purchasing Groups act to ensure that women with suspected ovarian cancer are referred to gynaecological oncologists or those gynaecologists who have the interest and the expertise to treat them correctly within a multidisciplinary setting.

*Received 9 March 1999*
*Accepted 16 March 1999*

*Correspondence to*: KK Chan

### REFERENCES

Adams M, Calvert AH, Carmichael J, Clark PI, Coleman RE, Earl HM, Gallagher CJ, Ganesan TS, Gore ME, Graham JD, Harper PG, Jayson GC, Kaye SB, Ledermann JA, Osborne RJ, Perren TJ, Poole CJ, Radford JA, Rustin GJS, Slevin ML, Smyth JF, Thomas H and Wilkinson PM (1988) Chemotherapy for ovarian cancer – a consensus statement on standard practice. *Br J Cancer* **78**: 1404–1406

Calman and Hine (1995) *A Policy Framework for Commissioning Cancer Services. Guidance for Purchasers and Providers of Cancer Services*. Department of Health: London

Faught W, Lotocki RJ, Heywood M and Krepart GV (1996) Early ovarian cancer. Value of a negative staging laparotomy. *Eur J Gynaecol Oncol* **17**: 200–203

Hawkins RE (1988) Chemotherapy for ovarian cancer – trials, controversies and funding. *Br J Cancer* **78**: 1402–1403

Junor EJ, Hole DJ and Gillis CR (1994) Management of ovarian cancer. Referral to a multidisciplinary team matters. *Br J Cancer* **70**: 363–370

Mayer AR, Chambers SK, Graves E, Holm C, Tseng PC, Nelson BE and Schwartz PE (1992) Ovarian cancer staging: does it require a gynecologic oncologist? *Gynecol Oncol* **47**: 223–227

Nguyen HN, Averette HE, Hoskins W, Penalver M, Sevin B-U and Steren A (1993) National Survey of Ovarian Cancer Part V: The impact of physician's speciality on patients' survival. *Cancer* **72**: 3663–3370

Petru E, Lahousen M, Tamussino K, Pickel H, Stranzl H, Stettner H and Winter R (1994) Lymphadenectomy in stage I ovarian cancer. *Am J Obs Gynecol* **170**: 656–662

Sandercock J, Parmar MKB and Torri V (1998) First-line chemotherapy for advanced ovarian cancer: paclitaxel, cisplatin and the evidence. *Br J Cancer* **78**: 1471–1478

Schueler JA, Trimbos JB, Hermans J and Fleuren GJ (1998) The yield of surgical staging in presumed early stage ovarian cancer. Benefits or doubts? *Int J Gynecol Cancer*. **8**: 95–102

Scott JS (1991) *Management of Ovarian Cancer*. *Current Clinical Practices*. *Report of a Working Group*. Standing Subcommittee on Cancer of the Standing Medical Advisory Committee: London

*© 1999 Cancer Research Campaign*

